# Availability of preoperative neutrophil-lymphocyte ratio to predict postoperative delirium after head and neck free-flap reconstruction: A retrospective study

**DOI:** 10.1371/journal.pone.0254654

**Published:** 2021-07-13

**Authors:** Hirotaka Kinoshita, Junichi Saito, Daiki Takekawa, Tasuku Ohyama, Tetsuya Kushikata, Kazuyoshi Hirota

**Affiliations:** Department of Anesthesiology, Hirosaki University Graduate School of Medicine, Hirosaki, Aomori, Japan; University of Florida, UNITED STATES

## Abstract

Postoperative delirium (POD) is a well-recognized postoperative complication and is associated with increased morbidity and mortality. We investigated whether the preoperative neutrophil-lymphocyte ratio (NLR) could be an effective predictor of POD after head and neck free-flap reconstruction. This was a single-center, retrospective, observational study. We analyzed the perioperative data of patients who had undergone elective head and neck free-flap reconstruction surgery. POD was assessed with the Intensive Care Delirium Screening Checklist (ICDSC) during admission to our intensive care unit (ICU). POD was defined as an ICDSC score ≥4. Risk factors for POD were evaluated by univariate and multivariate logistic regression analysis. We included 97 patients. The incidence of POD was 20.6% (20/97). Significantly longer ICU stays were observed in the patients with POD compared to those without POD (median [interquartile range]: 5 [4–6] vs. 4 [4–5], p = 0.031). Higher preoperative NLR values (3 <NLR ≤4 and 4 <NLR) were significantly associated with higher ICDSC scores compared to NLR ≤1 (4 [2–4] vs. 1 [1–1], p = 0.027 and 4 [1–4] vs. 1 [1–1], p = 0.038, respectively). The multivariable logistic regression analysis revealed that only a preoperative NLR >3.0 (adjusted Odds Ratio: 23.6, 95% Confidence Interval: 6.6–85.1; p<0.001) was independently associated with POD. The multivariate area under the receiver operator curve was significantly greater for the E-PRE-DELIRIC model with NLR compared to the E-PRE-DELIRIC model (0.87 vs. 0.60; p<0.001). The preoperative NLR may be a good predictor of POD in patients undergoing head and neck free-flap reconstruction.

## Introduction

Postoperative delirium (POD) is one of the most common postoperative complications. POD is associated with a decreased quality of life, prolonged hospital length of stay, and increased morbidity and mortality after major surgery [1]. The signs and symptoms of POD typically develop within the first 72 hr after surgery and last for several days, with a minority of cases persisting as cognitive dysfunction [2].

The reported incidence of POD after head and neck surgery ranges from 11% to 26% [3, 4]. Preventing POD in patients undergoing head and neck reconstruction surgery is important because POD can affect the flap blood flow and contribute to the development of postoperative cognitive dysfunction. Postoperative mechanical strain can cause a thrombosis in a flap, leading to flap necrosis. However, few established predictors for POD have been outlined.

The Early Prediction Model for Delirium in an intensive care unit (ICU) (E-PRE-DELIRIC) was proposed as a model for the early prediction of delirium of patients in the ICU [5]. Although the E-PRE-DELIRIC model is the most likely to be implemented successfully in daily ICU practice, this model has several limitations including low sensitivity and the necessity of additional calculation when the model predicts low risk. In addition, a recent study in a large United Kingdom hospital have shown that the utility of E-PRE-DELIRIC model was not as well calibrated as previously reported [6]. More highly sensitive predictive models for POD are urgently required to improve patients’ outcomes.

Both inflammation and oxidative stress may be involved in the pathophysiology of delirium [7]. The neutrophil-lymphocyte ratio (NLR), derived directly from the differential white blood cell (WBC) count, is an easily applicable marker of inflammation and oxidative stress. Several studies have reported an association between an increased NLR and poor prognosis of various cardiovascular diseases [8, 9] and malignancies [10]. A pilot study showed that the NLR was increased in acutely ill patients with delirium [11], and several studies showed that a higher preoperative NLR may be associated with the development of POD in patients undergoing cardiac surgery [12], carotid endarterectomy [13], and total hip arthroplasty [14]. In contrast, Ida and colleagues did not find a significant association between POD and the preoperative NLR [15]. There have been few investigations of the ability of the NLR to predict POD after surgery for a malignant tumor (including head and neck cancer).

We conducted the present study to determine whether the preoperative NLR can serve as an effective predictor of POD after head and neck free-flap reconstruction, and we compared the predictive performance between the preoperative NLR and the E-PRE-DELIRIC model.

## Materials and methods

### Study procedure and patients

This study adhered to the applicable STROBE statement. This single-center, retrospective, observational study was approved by the Ethics Committee of Hirosaki University Graduate School of Medicine, Hirosaki, Japan and was publicized on our department homepage (2020–158). The requirement for written informed consent was waived by the Ethics Committee because the study was retrospective and did not expose the patients to risk by an additional intervention. We enrolled 100 patients who underwent free-flap reconstruction of the head and neck following oncological surgery at Hirosaki University Hospital between January 1, 2015 and December 31, 2019. The exclusion criteria were: (1) prior diagnosis of preoperative dementia; (2) prior diagnosis of a neurologic disease such as Parkinson disease; and (3) a history of mental disorder such as major depressive disorder and schizophrenia.

### Data collection

Patient characteristics and perioperative data were obtained from our hospital electronic medical and anesthesia records. The patient characteristics included sex, age, body mass index (BMI), American Society of Anesthesiologist physical status (ASA-PS), comorbidities, diagnosis, pre-operative anticancer therapy, and TNM classification of malignant tumors. The severity of comorbidities on admission was calculated using the age-adjusted Charlson Comorbidity Index (ACCI) [16]. The ACCI is a validated tool enabling clinicians to predict post-treatment adverse events. The total ACCI score is obtained by adding the score given for age and each comorbidity; myocardial infarction, congestive heart failure, cerebral vascular disease, peripheral vascular disease, dementia, chronic obstructive pulmonary disease, connective tissue disease, peptic ulcer disease, mild liver disease, diabetes, diabetes with end-organ damage, hemiplegia, moderate/severe renal disease, any solid tumor, leukemia, lymphoma, moderate/severe liver damage, metastatic solid tumor, and acquired immunodeficiency syndrome.

The perioperative data included intraoperative fluid volumes, intra-operative blood loss, the use of allogeneic blood transfusion (red blood cell [RBC], fresh frozen plasma and platelet concentration), and the durations of surgery and anesthesia. The postoperative data also included postoperative doses of fentanyl and ketamine, and the postoperative incidence of metabolic acidosis, stroke, and myocardial infarction. Nine delirium predictors (age, history of cognitive impairment, history of alcohol abuse, blood urea nitrogen, admission category, urgent admission, mean arterial blood pressure, use of corticosteroids, and respiratory failure) were collected in consecutive patients immediately after ICU admission to calculate the E-PRE-DELIRIC score. The postoperative data including graft failure, length of stay (LOS) in the ICU, and LOS in the hospital were also collected. We divided the patients into two groups according to the development of POD: the POD group and the Non-POD group.

### Anesthesia and postoperative intensive care

All surgeries were conducted under total intravenous anesthesia (TIVA) and standard monitoring with electrocardiography and measurements of the peripheral oxygen saturation, end-tidal carbon dioxide concentration, direct arterial blood pressure, central venous pressure, body temperature (rectum), electroencephalography using the bispectral index (BIS), and urinary output. TIVA was induced and maintained with propofol, ketamine, remifentanil, and rocuronium bromide. Fentanyl and/or morphine were administered to relieve postoperative pain and stress. The depth of general anesthesia was adjusted to prevent the appearance of burst and suppression in the electroencephalogram: the target range of BIS was 40–60.

After the patients were transferred to the ICU following the surgery, propofol and dexmedetomidine were given at 2–3 mg/kg/hr and 0.2–0.7 μg/kg, respectively until the following morning. The patients received fentanyl and ketamine for pain control when necessary. The sedation and pain control were adjusted within Richmond Agitation Sedation Scale (RASS) −4 to −5 until the following morning to prevent mechanical strain to the free flap caused by spontaneous movements. In the morning of post-operative day 1, if the following were accomplished, the patient was converted from mechanical ventilation to t-piece oxygen therapy: PaO_2_/F_I_O_2_ >300 and the respiratory rate 12–20/min under positive end-expiratory pressure <5 cmH_2_O, stable hemodynamics in the range of heart rate < 40 beats per minute, systolic blood pressure > 80 and <180 mmHg, and no signs of increased work of breathing or distress were observed, e.g., accessory muscle use, paradoxical or asynchronous rib cage-abdominal movements, intercostal retractions, nasal flaring, profuse diaphoresis, and agitation. The subsequent sedation control was adjusted within RASS –1 to –2.

### Delirium assessment

We used the Intensive Care Delirium Screening Checklist (ICDSC) to screen all patients for delirium. The ICDSC was performed every 4 hr by one of the team of nurses, anesthesiologists, and intensivists. An ICDSC score ≥4 points indicates delirium [17]. The patients who had at least one positive POD diagnosis after their surgery were included in the POD group.

### Statistical analyses

The patients’ preoperative NLR values were divided into five groups: NLR≤1, 1<NLR≤2, 2<NLR≤3, 3<NLR≤4, and 4<NLR. The Kruskal-Wallis test and Dunn’s post hoc test were conducted to determine the association between the preoperative NLR and the maximum ICDSC score during the ICU stay.

We conducted a multivariate logistic regression analysis to identify predictive factors of POD. All continuous variables were converted to dichotomous variables in the multivariate analysis. The categorical data are presented as numbers (%). A receiver operating characteristic (ROC) curve analysis was performed to determine the best cut-off value of each type of continuous data including the preoperative NLR for predicting POD. Fisher’s exact test was used for the univariate analysis of all variables.

First, the NLR, the E-PRE-DELIRIC score, and the ACCI value were used as forced explanatory variables in the first multiple logistic regression analysis to evaluate the impact of these variables on the incidence of POD. We used the ACCI value as a covariate in our model to adjust for preoperative confounding factors. Generally, the number of events per predictor variable in a multivariate logistic should be ≥10 to provide an adequate predictive model [18]. However, Vittinghoff et al. suggested that 5–9 events per predictor variable were sufficient [19]. In our study, considering the number of events, three variables were included in the first models. We then included all variables with p<0.2 in the univariate analysis in the second multivariate model to identify all explanatory variables for POD.

Multicollinearity between two predictors was assessed by using the variance inflation factor (VIF). When the VIF was >10, one of two factors was deleted from the final model due to the multicollinearity. Discrimination was measured using the area under curve (AUC). The results are expressed as the adjusted odds ratios (ORs) with corresponding 95% confidence intervals (CIs). We performed a multivariate ROC curve analysis to verify whether the E-PRE-DELIRIC score along with the preoperative NLR could improve the predictive performance.

All statistical analyses were performed using EZR (Saitama Medical Center, Jichi Medical University, Saitama, Japan). A p-value <0.05 was considered significant in all tests.

## Results

### Incidence of POD

**Fig 1:**
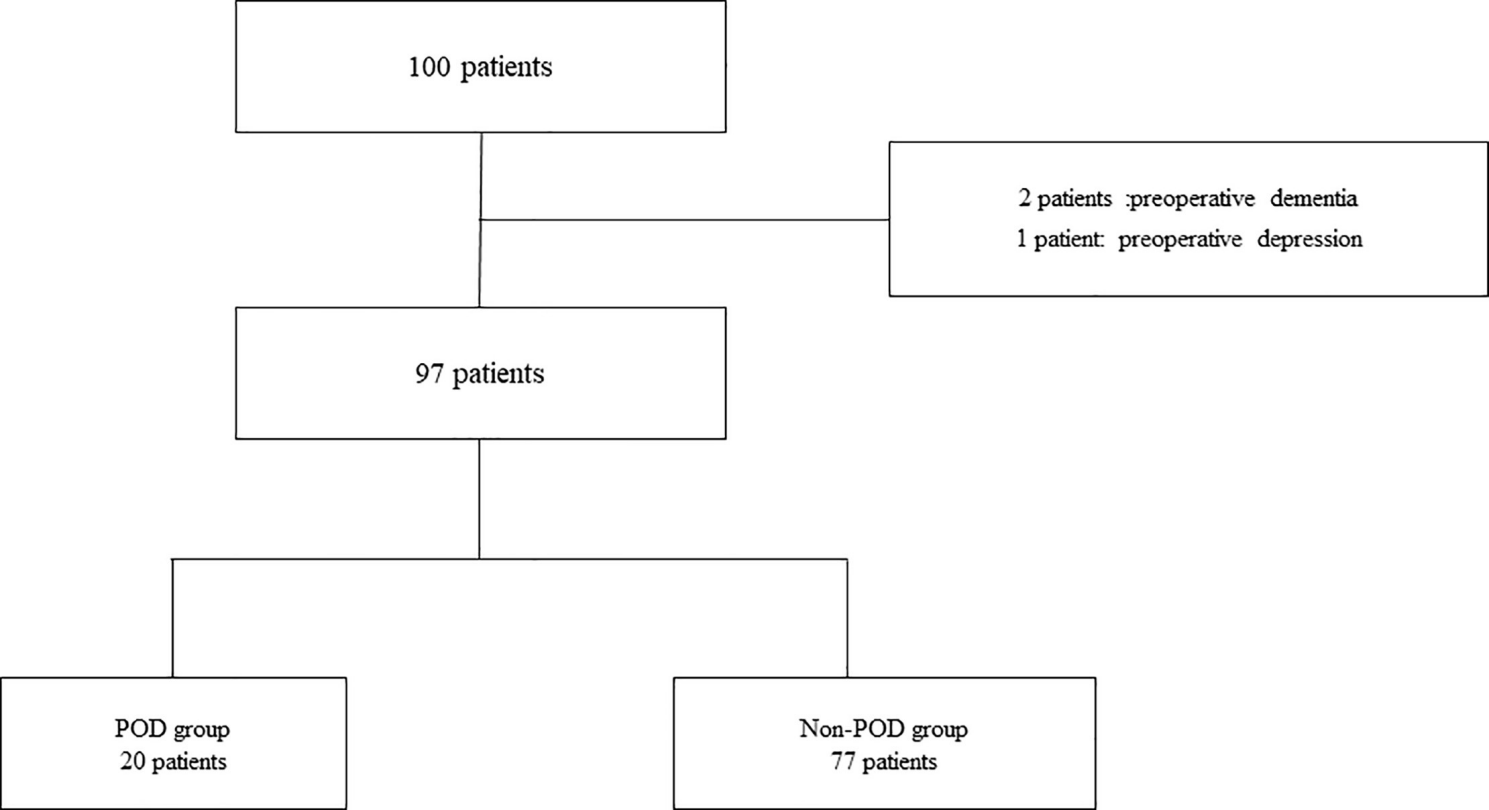
Study population flowchart. POD: Postoperative delirium.

The data described above were obtained from 100 patients, of whom three patients were excluded from the analysis due to exclusion criteria (dementia: 2, depression: 1). The cases of the remaining 97 patients were analyzed (Fig 1). Of the 97 patients, 20 patients were diagnosed with POD, and the 77 patients comprised the Non-POD group.

### Characteristics of the patients

The univariable analysis indicated that the preoperative NLR was significantly higher in the POD group: 4.24 [3.09–5.15] vs. 1.87 [1.46–2.49], p<0.001. The percentage of patients with a preoperative NLR >3.0 was significantly higher in the POD group compared to the Non-POD group: 15 (75.0%) vs. 9 (11.7%); p<0.001 (Table 1). There were significant differences in the BMI, the percentage of chronic obstructive pulmonary disease (COPD), and postoperative fentanyl dose between the two groups (Table 1). There were no significant differences in the other background characteristics.

**Table 1:** Patient characteristics and perioperative data in the patients with or without POD.

		POD	Non-POD	p-value
		n = 20	n = 77	
Male, n (%)		16 (80.0)	49 (63.6)	0.263
Age ≥73 yrs, n (%)		11 (55.0)	23 (30.0)	0.063
BMI ≤21 kg/m^2^, n (%)		13(65.0)	29 (37.7)	0.042^※^
ASA-PS, n (%)	1–2	12 (60.0)	62 (80.5)	0.076
	3	8 (40.0)	15 (19.5)	
Preoperative comorbidities, n (%)	Diabetes mellitus	5 (25.0)	12 (15.6)	0.511
	Hypertension	10(50.0)	(49.4)	>0.999
	Dyslipidemia	2 (10.0)	9 (11.7)	>0.999
	Chronic obstructive pulmonary disease	6 (30.0)	5 (6.5)	0.011^**※**^
	Atrial fibrillation	0 (0.4)	0 (0.0)	>0.999
	Ischemic heart disease	1 (5.0)	2 (2.6)	>0.999
	Stroke	2 (10.0)	5 (6.5)	0.631
	Chronic kidney disease	6 (30.0)	15 (19.5)	0.363
ACCI ≥5, n (%)		11 (55.0)	25 (32.5)	0.074
E-PRE-DELIRIC ≥0.8, n (%)		11 (50.0)	26 (33.8)	0.126
Preoperative anticancer therapy, n (%)	NAC	2 (10.0)	13 (16.9)	0.681
	PRT	5 (25.0)	9 (11.7)	0.249
Type of cancer, n (%)	Oral cavity	16 (80.0)	64 (83.1)	0.484
	Oropharynx	0 (0)	4 (5.2)	
	Hypopharynx	3 (15.0)	5 (6.5)	
	Others	1 (5.0)	4 (5.2)	
T stage, n (%)	I or II	9 (45.0)	42 (54.5)	0.610
	III or IV	11 (55.0)	35 (45.5)	
N stage, n (%)	0	8 (40.0)	42 (54.5)	0.568
	I	4 (20.0)	14 (18.2)	
	II	8 (40.0)	20 (26.0)	
	III	0 (0)	1 (1.3)	
M stage, n (%)	0	20 (100)	77 (100)	>0.999
Preoperative laboratory data, n (%)	NLR ≥ 3.0	15 (75.0)	9 (11.7)	<0.001^**※**^
Intraoperative factors n (%)	Duration of surgery, hrs, ≥12.0	8 (40.0)	22 (28.6)	0.416
	Duration of anesthesia ≥13.0, hrs	9 (45.0)	28 (36.4)	0.606
	Estimated blood loss ≥6.0, ml/kg/hr	16 (80.0)	50 (64.9)	0.283
	Blood transfusion	8 (40.0)	17 (22.2)	0.149
	Infusion volume ≤7.5, ml/kg/hr	12 (60.0)	36 (46.8)	0.325
Postoperative factors n (%)	Fentanyl dose ≥0.38 μg/kg/hr	9 (45.0)	12 (15.6)	0.016^**※**^
	Ketamine dose ≥ 5mg/day	16 (80.0)	56 (72.7)	0.58
	Metabolic acidosis	1 (5.0)	4 (5.2)	>0.999
	Stroke	0 (0.0)	0 (0.0)	>0.999
	Myocardial infarction	0 (0.0)	0 (0.0)	>0.999

Differences between the POD and Non-POD groups were estimated using Fisher’s exact test for all variables. Data are presented as number (percentage of each group). ACCI: Age-adjusted Charlson Comorbidity Index Hb: Hemoglobin, ASA-PS: American Society of Anesthesiologist-physical status, BMI: Body mass index, NAC: Neoadjuvant chemotherapy, NLR: Neutrophil-to- lymphocyte ratio, Plt: Platelet count, PRT: Preoperative radiotherapy, WBC: White blood cells.

### Clinical outcomes

The incidence of graft failure after surgery was similar in the two groups (Table 2). Significantly longer LOS in the ICU was observed in the patients with POD: 4 (4–5) days vs. 5 (4–6) days, p = 0.031. The LOS in the hospital tended to be longer in the POD group, but the difference was not significant (p = 0.072).

**Table 2:** Univariate analysis for clinical outcomes between POD group and Non-POD group.

	POD	Non-POD	p-value
	n = 20	n = 97	
Length of stay in ICU, days	5 [4,6]	4 [4,5]	0.031*
Length of stay in hospital, days	55 [44.8, 119.3]	46 [37.0, 61.0]	0.072
Flap failure n, (%)	3 (15.0)	15 (15.5)	0.758

The data are presented as numbers (%) or median values [25th–75th percentile]. ICU: Intensive care unit.

*p<0.05.

### Predictive models for POD

**Fig 2:**
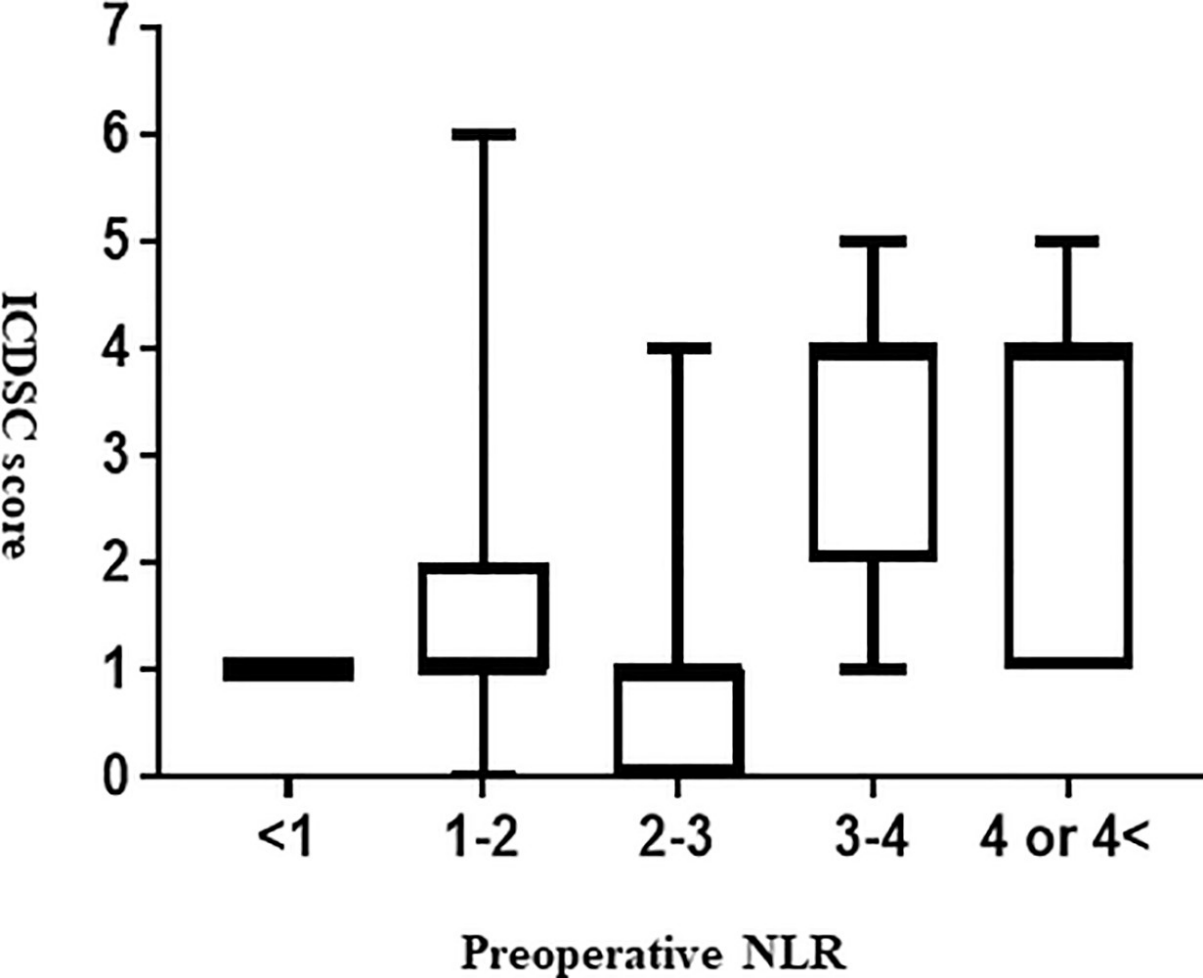
The association between the preoperative NLR and the maximum ICDSC score. Kruskal-Wallis test and Dunn’s post hoc test were used to determine the association between the preoperative NLR and the maximum ICDSC score. NLR: Neutrophil-to-lymphocyte ratio, ICDSC: Intensive Care Delirium Screening Checklist. *p<0.05 vs. NLR<1 Dunn’s post hoc tests.

Compared to NLR values ≤1, higher NLR values (3<NLR≤4 and 4<NLR) were associated with significantly higher ICDSC scores (p = 0.027 and p = 0.038, respectively) (Fig 2). We used the ACCI value as a covariate in our model to adjust for pre-operative confounding factors including age and COPD. The first multivariable logistic regression analysis revealed that a preoperative NLR >3.0 was independently associated with POD (OR: 13.80, 95%CI: 4.20–45.20; p<0.001) (Table 3). An E-PRE-DELIRIC score >0.08 (OR: 1.86, 95%CI: 0.46–7.42; p = 0.38) and an ACCI value >5.0 (OR: 2.05, 95%CI: 0.51–8.17; p = 0.31) were not associated with POD in the first multivariate analysis. The AUC value was 0.805 (95%CI: 0.683–0.927; p<0.05).

**Table 3:** Multivariate logistic regression analysis results with three variables.

	OR	95%CI	p-value
NLR >3.0	23.60	6.57–85.10	<0.001*
E-PRE-DELIRIC SCORE >0.08	1.86	0.46–7.42	0.379
ACCI >5.0	2.05	0.51–8.17	0.307

The AUC value was 0.85 (95%CI: 0.74–0.96). All VIFs were <10.

*p<0.05.

We conducted another multivariable logistic regression analysis that included all factors with p<0.2 in the univariate analysis. We thus included postoperative fentanyl dose ≥0.38 μg/kg/hr, BMI ≤21 kg/m^2^, ASA-PS 3, and blood transfusion during surgery in addition to the preoperative NLR and E-PRE-DELIRIC score in the models. The secondary multivariable logistic regression analysis revealed that a preoperative NLR >3.0 (OR: 38.4, 95%CI: 7.01–220.00; p<0.001) was independently associated with POD (Table 4). An E-PRE-DELIRIC score >0.08 (OR: 2.13, 95%CI: 0.46–9.91; p = 0.34) was not associated with POD as in the first multivariate analysis. The AUC value was 0.905 (95%CI: 0.818–0.992; p<0.05).

**Table 4:** Multivariate logistic regression analysis results.

	OR	95%CI	p-value
NLR >3.0	38.4	7.01–220.00	<0.001*
E-PRE-DELIRIC SCORE >0.08	2.13	0.46–9.91	0.337
ACCI >5.0	3.88	0.73–20.6	0.112
Postoperative fentanyl dose ≥0.38 μg/kg/hr	1.88	0.40–8.99	0.427
BMI <21 kg/m^2^	2.06	0.50–8.44	0.317
ASA-PS 3	0.23	0.03–1.55	0.130
Blood transfusion	1.64	0.36–7.45	0.523

The AUC value was 0.88 (95%CI: 0.79–0.98). All VIFs were <10. ACCI: Age-adjusted Charlson Comorbidity Index, ASA-PS: American Society of Anesthesiologist-physical status.

*p<0.05.

The AUC of the multivariate ROC was significantly greater for the E-PRE-DELIRIC model with the NLR compared to the E-PRE-DELIRIC model alone (0.87 vs. 0.60; p<0.001). The AUC of the multivariate ROC was also greater for the E-PRE-DELIRIC model with the NLR compared to the NLR alone model, there was not significantly difference between these two models (0.87 vs 0.84, p = 0.256).

## Discussion

### Main findings

The present results indicated that the preoperative NLR may be an independent predictable marker for POD after head and neck free-flap reconstruction surgery. The percentage of preoperative NLR values >3.0 in the POD group was significantly higher than that in the Non-POD group (15 [75.0%], 9 [11.7%]; p<0.01). Higher NLR values (3<NLR≤4 and 4<NLR) were significantly associated with a higher ICDSC score compared to NLR≤1 (p = 0.027 and p = 0.038, respectively). In addition, our multivariate logistic regression analysis showed that an NLR >3.0 was independently associated with POD. The NLR + E-PRE-DELIRIC model showed a better predictive performance compared to the E-PRE-DELIRIC model. To the best of our knowledge, this is the first study to determine the efficacy of the preoperative NLR for predicting POD after head and neck free-flap reconstruction surgery.

### Comparison with previous studies

#### POD and clinical outcomes

The overall incidence of POD in our study was 20.6%, which is comparable to the reported range of 11%–26% [7, 8]. Preventing POD in patients undergoing head and neck reconstruction surgery is important for the flap blood flow. Cervical kink, vasospasm, external compression, and thrombosis cause flap complications [20]. POD may be a risk factor for flap loss [21]. In our study, the rate of flap failure was not significantly different between the POD and Non-POD groups. The causes of flap loss are complex and include not only cervical restless due to POD but also optimal control of blood pressure. Notably, the present POD group may have included 10 patients with the hypoactive type of delirium, and the hypoactive type may have less effect on the flap’s condition.

Our analyses revealed longer LOS in the ICU in the patients with POD. Delirium in the ICU was associated with higher 6-month mortality and longer lengths of stay [22]. Prolonging the LOS in the ICU as a result of POD leads to delays in the patients’ ability to get out of bed and recover their nutritional status. Methods to prevent and treat POD are thus needed to change patients’ clinical outcomes including mortality, length of stay, cost of care, and cognitive function.

#### Predictive model for POD

Our findings indicate that the preoperative NLR may be an independent predictable marker for POD after head and neck free-flap reconstruction surgery. A few studies have described an association between the NLR and POD, and our findings are in agreement. A small pilot study revealed that an increased NLR in acutely ill patients was associated with delirium [10], and it was proposed that the preoperative NLR can be regarded as a potential marker for the prediction of early-onset delirium after acute ischemic stroke [23]. A prospective multicenter observational study revealed that a higher preoperative NLR was an independent predictor of POD in patients who underwent total hip arthroplasty [14]. Two observational studies regarding delirium after cardiac surgery showed that an increased postoperative NLR was associated with POD [12, 13]. These results suggested that systemic inflammation might reflect neuroinflammation and that both pre- and postoperative neuroinflammation could affect the development of POD.

In various stressful situations, the physiological response of the immune system is characterized by an increase in neutrophils and a decrease in lymphocytes [24, 25]. A high NLR was demonstrated to be an independent predictor of disease severity and poor prognosis. The preoperative NLR is a well-known prognostic marker for various cancers [26, 27]. Miyamoto et al. reported that the preoperative NLR is an additional useful predictor of both long-term and short-term outcomes in gastric cancer patients [28]. The NLR has also been shown to predict cardiac arrhythmias as well as short- and long-term mortality in patients with acute coronary syndrome [29]. A higher NLR was associated with frequent congestive heart failure decompensation and long-term mortality.

Our present analyses revealed that higher NLR values (3<NLR≤4 and 4<NLR) were significantly associated with higher ICDSC scores compared to NLR≤1 (p = 0.0265 and p = 0.0376, respectively), indicating correlations between the degree of preoperative inflammation and oxidative stress and the severity of POD. To the best of our knowledge, this is the first study to investigate the association between the preoperative NLR value and ICDSC score.

We observed that the NLR+ E-PRE-DELIRIC model showed a better predictive performance compared to the E-PRE-DELIRIC model. The E-PRE-DELIRIC model was proposed as an early prediction model for delirium in the ICU [5]. Although the E-PRE-DELIRIC model is likely to be implemented most successfully in daily ICU practice, this model has the limitations of low sensitivity and the necessity of PRE-DELIRIC calculation when the E-PRE-DELIRIC model predicts low risk. The area under the receiver operating characteristic curve was significantly greater for PRE-DELIRIC (0.74, 95%CI: 0.71–0.76) compared to E-PRE-DELIRIC (0.68, 95%CI: 0.66–0.71; z score of −2.73, p<0.01). There have been no studies of E-PRE-DELIRIC for the prediction of POD after head and neck free-flap reconstruction. A retrospective study in United Kingdom have shown that E-PRE-DELIRIC model was not as discriminative, or as well calibrated as previously reported [6]. We need highly sensitive predictive models for POD to stratify patients before surgery. The AUC of the present multivariate ROC was significantly greater for the NLR + E-PRE-DELIRIC model compared to the E-PRE-DELIRIC model (0.87 vs. 0.60; p<0.001). In addition, the AUC of the multivariate ROC was also greater for the NLR + E-PRE-DELIRIC model compared to the NLR alone model, but there was not significantly difference between these two models (0.87 vs. 0.84; p = 0.256). With the use of the NLR + E-PRE-DELIRIC model, we may identify patients who are at high risk of POD at the time of their admission to the ICU.

Many POD prediction models have been validated, but most models including E-PRE-DELICIC were static, making predictions based on data collected at a single time-point [30]. Considering the pathophysiology of critical illness and POD, future studies should be performed with dynamic indicators. NLR, which reflects the severity of inflammation, could be a dynamic indicator for predicting POD.

In another study, risk factors for the development of POD after head and neck free-flap reconstruction surgery were age, male sex, increased operative time, advanced nodal disease, and tobacco use [31]. In our univariable analysis, there was a significant difference in the rate of COPD between the POD and Non-POD groups. Although we performed a multivariate analysis with the ACCI value, no comorbidities were identified as a significant risk factor.

### Clinical implications

The NLR, derived directly from the differential WBC count, is an easily applied marker of inflammation and oxidative stress. Inflammation plays a critical role in tumorigenesis [32]. Tumor-extrinsic inflammation is caused by many factors, including bacterial and viral infections, autoimmune diseases, obesity, tobacco smoking, asbestos exposure, and excessive alcohol consumption, all of which increase the cancer risk and stimulate malignant progression. In contrast, cancer-intrinsic or cancer-elicited inflammation can be triggered by cancer-initiating mutations and can contribute to malignant progression through the recruitment and activation of inflammatory cells [33]. Chronic inflammation derived from cancer may induce neuroinflammation. Microglia of patients with cancer as well as elderly patients could take on a primed phenotype, characterized by dystrophic morphology, a progressive accumulation of metabolic stress, increased cell-surface expressions of antigen recognition molecules, and an exaggerated inflammatory response to immune challenge [34]. Primed microglia show an increased production of proinflammatory cytokines in response to immune stimulation with a peripheral inflammatory challenge, and this mechanism induces POD [35]. Although the NLR may suggest a neuroinflammatory condition, the detailed pathogenesis and underlying mechanisms require further study.

### Study strengths and limitations

This study has several limitations. It was a single-center, retrospective, observational study, and the sample size was relatively small. However, the preoperative NLR was significantly associated with the ICDSC score in the ICU and the data showed that the NLR plus E-PRE-DELICIC was better at discrimination. Second, we might have missed cases of hypoactive delirium. The patients who underwent head and neck reconstruction had undergone a tracheotomy and could not express their emotions. Third, we did not evaluate the patients’ preoperative cognitive function with a cognitive test such as the Mini-Mental State Examination. Moreover, we did not evaluate the validity of pain control.

Nevertheless, this was the first study to evaluate the association between the preoperative NLR and POD among patients undergoing elective scheduled cancer surgery. In addition, our results revealed the association between the preoperative NLR and the ICDSC score. The preoperative stratification of high-risk patients based on high NLR values could lead to effective prevention therapy including prehabilitation (particularly physical exercise), which may reduce neuroinflammation [36, 37].

## Conclusions

Our study demonstrates that the preoperative NLR could be a predictor of POD after head and neck free-flap reconstruction. Further studies are required to determine whether the NLR can predict POD in patients undergoing other types of oncological surgery.

## Supporting information

S1 File (XLSX)

## Abbreviations

POD: Postoperative delirium
ICU: Intensive care unit
NLR: Neutrophil-lymphocyte ratio
WBC: White blood cell
BMI: Body mass index
ASA-PS: American Society of Anesthesiologist physical status
ACCI: Age-adjusted Charlson Comorbidity Index
RBC: Red blood cell
LOS: Length of stay
TIVA: Total intravenous anesthesia
BIS: Bispectral index
RASS: Richmond Agitation Sedation Scale
ICDSC: Intensive Care Delirium Screening Checklist
ROC: Receiver operating characteristic
VIF: Variance inflation factor
AUC: Area under curve
ORs: Odds ratios
CIs: Confidence intervals
COPD: Chronic obstructive pulmonary disease
